# FIT for FUNCTION: study protocol for a randomized controlled trial

**DOI:** 10.1186/s13063-017-2416-3

**Published:** 2018-01-15

**Authors:** Julie Richardson, Ada Tang, Gordon Guyatt, Lehana Thabane, Feng Xie, Demetrios Sahlas, Robert Hart, Rebecca Fleck, Genevieve Hladysh, Louise Macrae

**Affiliations:** 1 School of Rehabilitation Science, McMaster University, Hamilton, ON Canada; 2 Department of Health Research Methods, Evidence, and Impact and Department of Medicine, McMaster University, Hamilton, ON Canada; 3 Centre for Evaluation of Medicine, St. Joseph’s Healthcare Hamilton, Hamilton, ON Canada; 4 Programs for Assessment of Technology in Health Research Institute, St. Joseph’s Healthcare Hamilton, Hamilton, ON Canada; 5 Department of Medicine, McMaster University, Hamilton, ON Canada; 6 Central South Regional Stroke Centre, Hamilton General Hospital, Hamilton Health Sciences, Hamilton, ON Canada; 7 Population Health Research Institute, Hamilton, ON Canada; 8 YMCA Hamilton, Burlington, Brantford, ON Canada

**Keywords:** Stroke, Community-based programs, Exercise, Function, Reintegration

## Abstract

**Background:**

The current state of evidence suggests that community-based exercise programs are beneficial in improving impairment, function, and health status, and are greatly needed for persons with stroke. However, limitations of these studies include risk of bias, feasibility, and cost issues.

**Methods/Design:**

This single-blinded, randomized controlled trial (RCT) of 216 participants with stroke will compare the effectiveness of a 12-week YMCA community-based wellness program (FIT for FUNCTION) specifically designed for community-dwelling persons with stroke to persons who receive a standard YMCA membership. The primary outcome will be community reintegration using the Reintegration to Normal Living Index at 12 and 24 weeks. Secondary outcomes include measurement of physical activity level using the Rapid Assessment of Physical Activity and accelerometry; balance using the Berg Balance Scale; lower extremity function using the Short Physical Performance Battery; exercise capacity using the 6-min walk test; grip strength and isometric knee extension strength using hand held dynamometry; and health-related quality of life using the European Quality of Life 5-Dimension Questionnaire. We are also assessing cardiovascular health and lipids; glucose and inflammatory markers will be collected following 12-h fast for total cholesterol, insulin, glucose, and glycated hemoglobin. Self-efficacy for physical activity will be assessed with a single question and self-efficacy for managing chronic disease will be assessed using the Stanford 6-item Scale. The Patient Activation Measure will be used to assess the patient’s level of knowledge, skill, and confidence for self-management. Healthcare utilization and costs will be evaluated. Group, time, and group × time interaction effects will be estimated using generalized linear models for continuous variables, including relevant baseline variables as covariates in the analysis that differ appreciably between groups at baseline. Cost data will be treated as non-parametric and analyzed using a Mann–Whitney U test.

**Discussion:**

This is a RCT with broad study eligibility criteria intended to recruit a wide spectrum of individuals living in the community with stroke. If positive benefits are demonstrated, results will provide strong research evidence to support the implementation of structured, community-based exercise and education/self-management programs for a broad range of people living in the community with stroke.

**Trial registration:**

ClinicalTrials.gov, NCT02703805. Registered on 14 October 2014.

**Electronic supplementary material:**

The online version of this article (doi:10.1186/s13063-017-2416-3) contains supplementary material, which is available to authorized users.

## Background

Stroke is the leading cause of neurological disability in adults, with direct and indirect costs in Canada amounting to $3.6 billion annually [1]. In 2013, we estimate that there were 405,000 individuals experiencing the effects of stroke in Canada, yielding a prevalence of 1.15%, and projected to increase to 726,000 by 2038 [2]. By 2021, approximately 6 million Canadians will be aged > 65 years, substantially increasing the burden of disability associated with stroke within the next decade. Cardiovascular disease (CVD) is the leading cause of death after stroke [3] and the risk for recurrent stroke is high: 26% at five years and 39% at ten years [4]. An estimated 17.3 million people died from CVD in 2008 and 6.2 million deaths were due to stroke [5].

Low levels of physical activity have a long-term impact on mobility and health outcomes by perpetuating sedentary behaviors, contributing to further mobility limitations, and elevating the risk for future events. Decreased motor performance [6–8] and physical inactivity [9] are also associated with dissatisfaction and lower quality of life (QOL) after stroke. This downward cycle may be interrupted through regular exercise and enhanced physical activity, but formal rehabilitation services are time-limited, often lasting only a few weeks [10], and rarely beyond one year [11]. Moreover, few opportunities exist for ongoing community exercise beyond rehabilitation [12] and many modifiable risk factors such as physical inactivity, cardiovascular disease, depression, and diabetes remain poorly managed after stroke [13, 14]. Innovative and sustainable community programs to optimize function and minimize burden and costs of stroke are urgently needed.

### Benefits of exercise after stroke

Persons with stroke need access to exercise programs where the benefits on motor control, strength, upper extremity function, mobility, balance, and aerobic capacity have been established [15–21]. However, often these programs are developed in the context of research projects and thus have time-limited accessibility [22]. A summary of these studies follows, with more detail provided in Additional file 1.

#### Functional benefits with community-based exercise after stroke

In pre-post intervention trials, community stroke exercise programs have been shown to improve muscle strength [21, 23, 24], gait speed [11, 21, 23, 24], balance ability [11, 22, 24, 25], timed up-and-go time [22], and 6-min walk test (6MWT) distance [25]. Noteworthy are two prospective trials of community-based stroke programs. First, a prospective cohort design compared the impact of a self-management and exercise program to an education program (Living with Stroke [LWS]). Increased social support was observed following both programs, but only the exercise intervention also improved community integration, and benefits were retained 12 weeks after the programs ended [26, 27]. Second, in one of the first randomized trials focusing on stroke-specific community-based exercise, the Fitness and Mobility Exercise (FAME) program was effective in improving aerobic capacity, gait speed, and paretic-side muscle strength, but no outcomes related to community reintegration or QOL were included, nor were follow-up assessments conducted [28].

#### Effects of post-stroke community exercise on cardiovascular risk factors

While benefits to cardiovascular risk factors have been demonstrated after exercise in other populations, few trials have examined the analogous effects of post-stroke exercise. Cardiovascular risk factors, associated with primary and secondary event risk, are prevalent after stroke [3]. High blood pressure (BP) is the strongest independent predictor for ischemic and hemorrhagic stroke [29] and interventions to lower BP are associated with lowered stroke risk [30]. Dyslipidemia is a major risk factor for coronary artery disease [31] and is associated with increased stroke risk [32, 33]. Inflammatory markers are elevated with age and in the presence of chronic conditions such as cardiovascular disease, hypertension, and diabetes, yet are inversely associated with physical activity and exercise in older adults [34, 35].

In the few studies that have examined the effects of exercise training on cardiovascular risk factors after stroke, inconsistent benefits have been reported and none have examined the retention of benefits. Training-related improvement in BP at submaximal workloads of exercise has been reported [20], but there are conflicting effects on lowered resting heart rate (HR) [20, 36]. Even fewer studies have examined the effects of community-based exercise programs on cardiovascular risk factors. Improvements in fasting glucose [37], insulin, and glucose tolerance and insulin sensitivity have been previously reported [34, 38]. Community-based cardiac rehabilitation models of care that specifically focus on exercise and secondary prevention have also been adapted successfully for people with moderate disability from stroke [39] with demonstrated improvements in a composite cardiac risk score, although not in individual risk factors such as waist circumference, cholesterol levels, BP, or aerobic capacity [40]. In individuals with little to no disability following stroke, improvements in aerobic capacity, lipid profiles, and body composition measures were observed [41].

#### Cost-effectiveness of community-based exercise programs

One of the aims of exercise programs is to increase overall physical activity levels to counteract potential negative health consequences that result from long-term sedentary behaviors. Cost-effectiveness analysis that jointly considers healthcare resource uses and effect on health-related QOL provides an important piece of information for policy making. A commonly used summary measure for cost effectiveness analysis is incremental cost per quality-adjusted life years (QALY) gained by a new intervention in comparison to its comparators. The cost-effectiveness of strategies to promote physical activity have been demonstrated in various groups [42] and high-risk populations [43–45]. However, such cost-effectiveness analysis for stroke-specific community exercise programs has not been previously studied.

### Need for effective and sustainable community stroke exercise programs

The current state of evidence suggests that community-based exercise programs are beneficial in improving impairment, function, and health status and are greatly needed for persons with stroke. However, limitations of these studies include risk of bias, feasibility, and cost issues. The majority were non-randomized trials with small samples that did not evaluate for retention of benefits. Many studies were conducted in laboratory settings, required health professionals for program delivery, and therefore less sustainable, and did not include participant-important outcomes, such as reintegration and QOL. Few community-based exercise trials have examined the effects on cardiovascular risk factors and no previous study has included cost or healthcare utilization analyses. Moreover, many studies were conducted in laboratory settings, required health professionals for program delivery and therefore less sustainable, and did not include participant-important outcomes, such as reintegration and QOL. A Canadian trial with a cost-effectiveness analysis is needed to evaluate an evidence-based intervention with an adequate follow-up.

From 2011–2012, we undertook a randomized controlled pilot study, funded by the Ontario Stroke Network, to establish the feasibility and preliminary effects of FIT for FUNCTION, a community-based wellness program for stroke survivors. This 12-week YMCA-based exercise and self-management education program was compared to a 12-week standard YMCA membership (without participation in a formal program) that served as the control intervention. An innovative feature of this intervention lies in the LiveWell Partnership between the YMCA, Hamilton Health Sciences, and McMaster University that crosses sectors to create sustainable evidence-based community programs [46]. The Les Chater YMCA in Hamilton, Ontario, Canada, was used for the site of this pilot study. The program was supported by a stroke rehabilitation physiotherapist from Hamilton Health Sciences and evaluation of program outcomes led by researchers at McMaster University. Sixty-one participants were evaluated pre and post intervention and at 24-week follow-up. As a result of this pilot study, we were able to confirm the feasibility of recruitment and randomization, and that stroke survivors could safely undertake this community-based exercise intervention. The FIT for FUNCTION group demonstrated greater improvement after the 12-week intervention compared to the control group with respect to community integration (Reintegration to Normal Living Index [RNLI]), balance (Berg Balance Scale [BBS]), lower extremity function (Short Physical Performance Battery [SPPB]), and walking ability (6MWT), which were maintained at 24-week follow-up. Specific measures of cardiovascular function were not included in the pilot study, nor were analyses of cost-effectiveness of the program.

To confirm our preliminary findings and to expand access to the FIT for FUNCTION program to more communities across south-central Ontario, we propose a larger-scale randomized controlled trial (RCT) to evaluate FIT for FUNCTION in three additional YMCA sites in south-central Ontario (Downtown Hamilton, Brantford, Niagara). These programs will be supported by stroke rehabilitation physiotherapists from Hamilton Health Sciences, Brantford Community Health System, and Niagara Health Care System. This evidence-based initiative has modest resource demands, capitalizes on existing local community program infrastructure, and will serve as a model for sustainable, accessible, effective, and cost-effective wellness programs for individuals with chronic conditions. This larger study is single-blinded in design, will include additional outcomes related to cardiovascular health and function to evaluate the program’s effectiveness on mediating cardiovascular risk, evaluate for retention of benefits following program completion, and examine the cost-effectiveness of this program.

### Objectives

The primary objective of this RCT is to assess the effectiveness of FIT for FUNCTION, a 12-week YMCA community-based wellness program specifically designed for people with stroke, vs a standard YMCA membership (no formal program) on community integration among people with stroke at 12 and 24 weeks. The secondary objectives are to evaluate the effect of FIT for FUNCTION program vs standard YMCA membership on physical activity levels, physical functioning, health-related QOL, cardiovascular health, and self-management. Tertiary objectives are to evaluate the retention of benefits, healthcare utilization and costs, cost-effectiveness, and to determine the prognostic indicators associated with program responsiveness.

#### Research questions and specific hypotheses

The proposed trial aims to answer the following research questions:

1. Is the 12-week FIT for FUNCTION stroke program more effective than a standard YMCA membership in improving community reintegration (primary outcome) at 12 and 24 weeks?

*Hypotheses:* Participants in the FIT for FUNCTION group will demonstrate greater post-program improvement in community integration. Gains will be maintained at 24 weeks (12 weeks following program completion).

2. Is the FIT for FUNCTION stroke program more effective than a standard YMCA membership in increasing levels of physical activity and improving physical functioning (walking, balance, strength), health-related QOL, cardiovascular health, and self-management at 12 and 24 weeks?

*Hypotheses:* Participants in the FIT for FUNCTION group will demonstrate greater post-program improvement in physical activity, physical functioning, health-related QOL, cardiovascular health, and self-efficacy. Gains will be maintained at 24 weeks (12 weeks following program completion).

3. Is the FIT for FUNCTION program more cost-effective than a standard YMCA membership?

*Hypotheses:* Participants in the FIT for FUNCTION program will have lower direct and indirect medical costs over the 24 weeks of the trial period as evidenced by fewer hospital and physician visits, and formal and informal caregiver services. The costs associated with the FIT for FUNCTION program will be less than those for the control group. We also hypothesize that the FIT for FUNCTION program will be more effective in improving health-related QOL of participants.

4. What participant characteristics are associated with degree of program response observed?

*Hypotheses:* Participants who demonstrate greater improvement in community integration (primary outcome) from the FIT for FUNCTION program will have less co-morbidities, illness severity, depressive symptoms, mobility, and cognitive impairment.

### Proposed subgroup analyses

We will also complete two subgroup analyses to address the following questions.

1. Are there sex differences in the specified outcomes? We will use an interaction term sex × treatment group to complete this analysis.
2. Are there differences in according to age in the specified outcomes? We will use an interaction term age (18–55 years) (56+ years) to complete this analysis.

## Methods/Design

### Research design

**Fig. 1:**
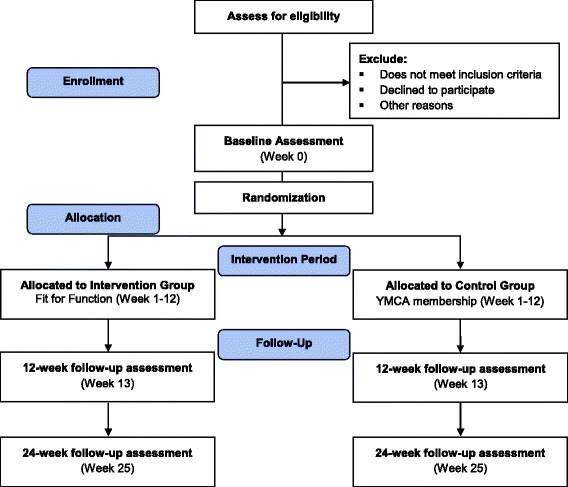
Study flow *diagram*

This is a RCT [47, 48], evaluating a complex intervention and reflecting the heterogeneity of individuals living in the community with stroke who might benefit from this intervention. The Standard Protocol Items: Recommendations for Interventional Trials (SPIRIT) checklist is available in Additional file 2. Both outcome assessors and data analysts will be blind to group allocation. Participants will be evaluated at three time points: baseline (0 weeks); at the end of the intervention (12 weeks); and at follow up (24 weeks) (Fig. 1).

#### Conceptual framework

We have used the International Classification of Functioning, Disability and Health (ICF) [49] as our conceptual framework to identify key factors associated with participation in community-based physical activity (see Additional file 3). The first component of the ICF (Impairments) will be used as covariates in analyses of research questions 1 and 2 [50]. The second component of the ICF (Activities and Participation) will be defined using relevant mobility, self-care measures, and participation measures. The concepts of our primary outcome measure, the Reintegration to Normal Living Index, have been mapped to the ICF [51]. Qualitative analyses will identify the environmental (e.g. services, systems, and policies) and personal factors (e.g. self-efficacy for physical activity) within the ICF framework that impact activities and participation.

#### Sites

The intervention will be implemented at three YMCA sites in south-central Ontario: Downtown Hamilton; Brantford; and Niagara. Ontario is the most populous province in Canada, representing 38% of the nation’s population [52] and the central-south region has the highest prevalence of stroke in the province [53]. The YMCA of Hamilton/Burlington/Brantford is the third largest YMCA in Canada, serving approximately 190,785 people in over five membership sites in the communities of Hamilton Downtown, Hamilton Mountain, Brantford, Flamborough and Burlington. The YMCA of Niagara is also one of the largest in Canada, with programs and services in more than five membership sites across the region.

#### Participant eligibility criteria

Eligible participants with stroke will be: age ≥ 18years; living in the community; able to independently ambulate ≥ 10 m with or without an assistive device; able to tolerate 60 min of activity with rest intervals; not actively engaged in active rehabilitation; able to independently follow instructions; and medically cleared from a physician to participate in the program. We will use the Montreal Cognitive Assessment (MoCA) to describe the level of cognitive impairment that would prevent the participant from independently learning and carrying out an exercise program. Participants will need to be able to communicate English and not have a comprehensive or global aphasia that would prevent them from learning and participating in the educational component of the intervention [54].

#### Recruitment strategy

We successfully enrolled 61 participants in the pilot study and a similar recruitment strategy will be used for the current study. Participants will be recruited by referrals from: (1) inpatient acute care, inpatient rehabilitation, outpatient rehabilitation, and stroke prevention clinics at the Regional Stroke Centre at Hamilton Health Sciences, Brantford Community Health System, and Niagara Health System; (2) family physicians who will be informed about the study through education events; and (3) through the Stroke Recovery Chapters, media community announcements, and through the YMCA website. Referrals will be made by phone to the Lead Research Coordinator (LRC) at McMaster University. Per pilot study protocol, the LRC will establish initial eligibility by phone, followed by a second in-person eligibility screening session at the baseline assessment. Signed medical clearance from the participant’s family physician will be obtained.

#### Randomization

The unit of randomization will be the participant and the randomization process will use computer generation of group assignment with a 1:1 allocation ratio. Participants will be stratified by site (three YMCA sites) and by walking ability (10-m walk speed ≤ 0.9 m/s [household or limited community ambulator] or > 0.9 m/s [unlimited community ambulator]) [55]. We will block the randomization with variable block size unknown to the sites. Once participant consent has been obtained and baseline assessment completed, the LRC will enter the Methods Centre at McMaster University to report the participant characteristics needed for stratification into REDCap, a secure web-based data management system, and receive the group allocation, thus ensuring concealment of randomization.

### Interventions

Participants will be randomized into either a group that will participate in FIT for FUNCTION or receive a standard YMCA membership. For both groups, attendance will be recorded, resting blood pressure will be taken, and a pre-exercise safety checklist will be reviewed before each exercise session.

#### Intervention group: FIT for FUNCTION

The 12-week FIT for FUNCTION program comprises an exercise component (2×/week group classes, 1×/week independent exercise) and a self-management / education component (1×/week).

1. Exercise: group exercise sessions (60 min, 2×/week) will be delivered by a kinesiologist trained to work with individuals with stroke. A stroke rehabilitation physiotherapist will provide on-site consultation 4 h per week and remote consultation 3 h per week. These group classes were adapted from the Community-Based Exercise Program for Persons Living with Stroke [56] and a DVD training package was developed for staff as a standardized competency-based approach. The exercise classes comprise: (i) warm up (10 min); (ii) task-oriented strengthening and cardiovascular conditioning (20 min); (iii) mobility and balance (20 min); and (iv) cool down (10 min). The feasibility of this program was established in the pilot study, with no adverse events and four participants (6.6%) withdrawing from the program (one related to the intervention and three unrelated to the intervention). From the pilot study, we found that maintaining a class size of 12 and a staff:participant ratio of 1:4 was important for ensuring safety of all participants. See Additional file 4 for a detailed description of the exercise program.

Of note, the cardiovascular conditioning component will be enhanced in this proposed trial. Individualized prescriptions will be determined using HR reserve and rating of perceived exertion methods that we have used extensively in previous stroke trials [37, 39]. Participants will also attend an independent exercise session (1×/week), where they will have full access to equipment and facilities at the YMCA.

For all exercise sessions, duration (time) and intensity (HR, rating of perceived exertion) will be recorded.

2. Self-management and education. LWS is an eight-session group self-management education program facilitated by a LWS-trained YMCA staff member, with the option of utilizing a stroke survivor as a co-facilitator. These sessions are held 1×/week and help stroke survivors and their caregivers cope with stroke related changes and engage as active participants in the management of their care. A self-management problem-solving approach is used to develop self-efficacy. Topics include: understanding stroke; physical changes and challenges; swallowing and nutrition; cognition, perception, and communication; emotions; focusing on depression; activities and relationships; reducing the risk of stroke; and moving forward.

#### Control group: standard YMCA membership

The control group will receive a 12-week complimentary YMCA membership and the frequency of attendance to engage in exercise will be self-determined. As part of this membership, participants will have access to eight fitness coaching sessions to receive instruction about the use of equipment or exercise regimes. These coaching sessions will be provided by YMCA staff who we will train to work with individuals with stroke but are not involved in the FIT for FUNCTION program. The standard YMCA membership was chosen as the control intervention since it reflects what is currently available as an option for people with stroke wanting to participate in community-based exercise programming in the region.

### Assessments

Before the initial assessment, the assessor will obtain written consent from each participant. Data will be entered directly into the database at the time of assessment using a tablet with a remote connection to REDCap, a secure web-based data management system. All assessments will take place at the YMCA with a physiotherapist trained in the administration of the outcome measures and in the use of REDCap.

**Fig. 2:**
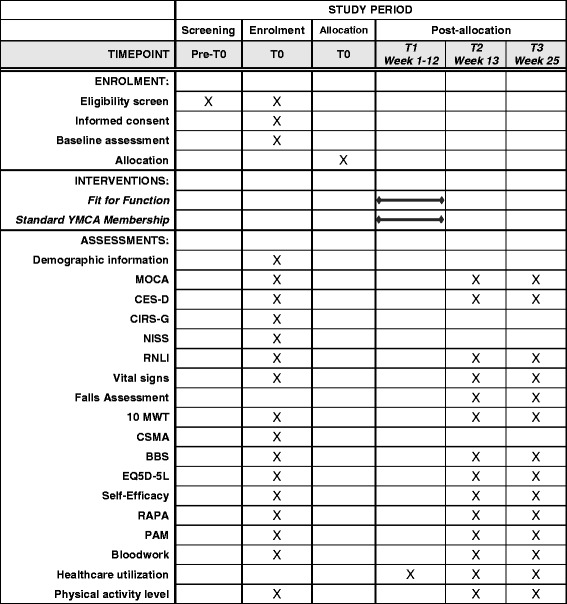
Schedule of enrolment, interventions, and assessments. MOCA Montreal Cognitive Assessment, CES-D Centre for Epidemiological Studies Depression Scale, CIRS-G Cumulative Illness Rating Scale for Geriatrics, NISS National Institutes of Health Stroke Scale, RNLI Reintegration to Normal Living Index, 10 MWT, Ten-meter walk test, CMSA Chedoke-McMaster Stroke Assessment, BBS Berg Balance Scale, EQ5D-5L European Quality of Life 5-Dimension Questionnaire, RAPA Rapid Assessment of Physical Activity, PAM Patient activation measure, Bloodwork Glucose, insulin, HbA1c, triglycerides, cholesterol, cholesterol/HDL ratio, non-HDL cholesterol, C-reactive protein

Results from the pilot study confirm that the assessment sessions took approximately 90 min per participant and the sessions were generally well tolerated. At baseline, participant demographics will be recorded, along with stroke severity (NIH Stroke Scale), stage of recovery (Chedoke-McMaster Stroke Assessment), and co-morbidity and illness severity (Cumulative Illness Rating Scale) [57]. Cognitive impairment (MoCA) [58], presence of depressive symptoms (Centre for Epidemiologic Studies Depression Scale) [59, 60], and self-reported falls will be documented at baseline, 12 weeks, and 24 weeks along with the assessment of the primary and secondary outcomes (Fig. 2).

### Primary outcome

#### Community reintegration

The RNLI assesses the degree to which individuals who have experienced traumatic or incapacitating illness achieve reintegration into normal social activities (e.g. recreation, movement in the community, and interaction in family or other relationships) [61]. It has good content validity (Cronbach’s Alpha [*r* > 0.90]) and sensitivity to change [62]. Seven out of 11 items showed substantial test–retest reliability (kappa > 0.61) [63].

### Secondary outcomes

#### Level of physical activity

The Rapid Assessment of Physical Activity (RAPA) is a nine-item measure that assesses frequency and intensity of aerobic physical activity and frequency of strengthening and flexibility exercises [64]. Compared to a criterion standard, the Community Healthy Activities Model Program for Seniors [64], the RAPA is more highly correlated (*r* = 0.54) with moderate caloric expenditure than the Patient-centered Assessment and Counselling for Exercise (*r* = 0.44) [65]. It has a reported sensitivity (81%) and positive predictive value (77%) [64].

We will also include accelerometry to quantify activity counts as an objective measure of habitual physical activity (Actical System, Philips Respironics, Bend OR, USA). The Actical is a small (29 × 37 × 11 mm), lightweight (16 g) omnidirectional accelerometer that measures activity counts via vibrations, which are converted into step counts and time spent in four activity levels [66]. It will be secured to the ankle using a nylon strap and worn for three consecutive days (from Friday to Sunday to capture weekdays and weekend days). The accelerometry data will form part of the more comprehensive profile of cardiovascular health that will capture physical activity levels, resting blood pressure and HR, lipid profiles, glucose, and inflammatory markers.

#### Balance

We will use the BBS to assess dynamic and static balance and risk of falls. It has excellent reliability for both acute stroke (intraclass correlation coefficient [ICC] = 0.95) [67] and chronic stroke (ICC = 0.98) [68]. In patients with stroke and residual gait deficits, the minimal clinically importance difference was detected as 5.8 points [69, 70].

#### Physical functioning

The SPPB will be used to quantify lower extremity function by evaluating the time to walk 8 feet, standing balance, and repeated chair stands. A validation study showed an association between the performance of each of these tests and self-reported disability [71]. The 6MWT provides an indicator of exercise capacity by measuring the distance walked in 6 min [72]. The test–retest reliability of this test has been established in people with stroke (ICC = 0.973) and the minimal detectable change is 54.1 m [73]. Several studies have demonstrated the responsiveness of 6MWT distance after gait-targeted interventions with individuals with stroke [15, 74, 75]. Resting HR and blood pressure as well as a rate of perceived exertion (RPE) and fatigue level (0–10) are measured before commencing the 6MWT. Grip strength and isometric knee extension strength will be measured using hand-held dynamometry. For both tests, the average of three trials will be calculated. Handgrip has been shown to correlate with elbow flexion strength, knee flexion strength, and trunk extension strength [76].

#### Health-related quality of life

The European Quality of Life 5-Dimension Questionnaire (EQ-5D-5L) is a generic utility-based health-related QOL questionnaire [77]. It consists of five questions covering the dimensions of mobility, self-care, usual activities, pain/discomfort, and anxiety/depression with five response options indicating no, mild, moderate, severe, and extreme problems for each dimension [78]. The EQ-5D-5L defines a total of 3125 health states which can be converted to a utility index anchored at 0 for a state being equal to dead and 1 for full health. The utility index combined with life expectancy allows for the estimation of QALY, a recommended generic outcome measure in health economic evaluation in Canada (Canadian Drug and Technology Agency, Ontario Ministry of Health and Long-Term Care) and other countries (National Institute for Clinical Excellence, Australia Department of Health) [79–82]. The EQ-5D-5L is a prominent example of generic utility-based questionnaire with some indicators showing it is the most widely used in health economic evaluation [83]. The EQ-5D-5L has been validated in individuals undergoing stroke rehabilitation [84].

#### Cardiovascular health

HR and BP will be measured after 5 min sitting at rest. BP will be measured at the brachial artery of the unaffected arm. Two measurements will be taken and averaged. If values differed > 5 mmHg, two more readings were done and the average taken across four readings [85]. HR and BP will also be measured at the end of the 6MWT as indicators of cardiovascular response during sub-maximal exercise. Body mass, height, and waist and hip circumference will be measured and body mass index and waist:hip ratio calculated. Blood samples will be collected following 12-h fast for lipid profile (total cholesterol, high-density lipoprotein cholesterol, low-density lipoprotein cholesterol, triglycerides), glucose (glucose, insulin, glycated hemoglobin), and inflammatory markers (C-reactive protein).

#### Self-efficacy

Self-efficacy (SE) for physical activity will be assessed with a single question. Participants will be asked to rate how confident they are that they could participate in moderate intensity physical activity for 150 min per week on a scale of 1 (not confident) to 10 (very confident) [86]. Self-efficacy for managing chronic disease will be assessed using the Stanford Self-Efficacy for Managing Chronic Disease 6-Item Scale. This six-item scale is less burdensome for patients, containing items taken from several SE scales developed for the Stanford Chronic Disease Self-Management Program [87].

#### Self-management

The Patient Activation Measure (PAM) is a 13-item measure of the patient’s level of knowledge, skill, and confidence for self-management [88]. Cronbach’s alpha = 0.87 and test–retest reliability have been reported as high [89]. PAM scores were compared to three judges’ classification of the individuals’ level of activation with kappa = 0.8–0.9 as evidence of validity [59]. Test–retest reliability has been reported as ICC = 0.85 [90].

#### Healthcare utilization and costs

The healthcare resource utilization will be evaluated using a case report form (CRF) specifically developed for the economic evaluation. Relevant healthcare resource utilization will include specialist visits, family physician visits, emergency room visits, hospitalization, and medication related to stroke over the 24-week trial period. These resource utilizations will be converted to direct medical costs using appropriate unit costs from available public databases such as Ontario Health Insurance Plan, physician fee schedule, and Ontario Case Costing Initiative. Other relevant direct non-medical costs include the time of the kinesiologist and fitness instructor who will provide group exercise sessions, the cost of delivering eight-session group self-management education program by LWS-trained YMCA staff, the cost of three-month YMCA membership, the cost of developing the education materials, and the cost of transportation to and from the YMCA. Indirect costs may include the time of informal caregiver if any to provide assistance to the participating patient.

### Qualitative methods

Individual interviews will be used to investigate the participant perceived impact of the intervention and to substantiate the quantitative results, helping to answer research questions 1 and 2. An independent, experienced facilitator will conduct the interviews, which will be recorded and transcribed verbatim. We have utilized interview results from the pilot study to make modifications to the program and its processes.

### Adverse events

The occurrence of adverse events will be monitored, including muscle stiffness, soreness, falls, or injuries resulting from the intervention. An Adverse Event Form will be completed by YMCA staff.

### Sample size justification

The sample size calculation is based on the test of the null hypothesis that the mean reductions in RNLI scores in the two populations (intervention and control) are equal. The primary measure of effect is the change in RNLI scores from baseline to 12 weeks (two-tailed type I error of 0.05; type II error of 80%). The criterion for significance (alpha) has been set at 0.05. The test is two-tailed, which means that an effect in either direction will be interpreted. The sample size of 105 per group was calculated using an a priori sample size calculator for Student’s t-tests [91]. With the proposed sample size of 105 in each of the two groups (i.e. assuming a 1:1 allocation ratio), the study will have power of 80% to yield a statistically significant result using a t-test (assuming an intention-to-treat principle for the analysis) of the difference between mean changes in RNLI scores (12 weeks to baseline) at alpha = 0.05. This computation assumes that RNLI change scores are normally distributed, the mean difference is 3.1 (95% confidence interval [CI] = -3.37–9.56) (corresponding to mean changes of 6.26 [for the intervention group] vs 3.16 [for the control group]) and the common within-group standard deviation is 3.23 These estimates are modified estimates from our pilot trial, which account for the type of intervention planned for in this study. We calculated the sample size to address the primary outcomes. Table 1 provides the sample sizes for different values of the expected plausible difference and standard deviations.

**Table 1:** Sample sizes for different plausible values of the mean difference and corresponding standard deviations

SD	1.0	2.0	3.0	4.0
3.0	292	76	38	20
4.0	506	128	60	24
5.0	788	200	90	52
6.0	1230	352	128	74
7.0	1604	404	182	100
8.0	1806	506	220	128
9.0	2598	652	292	200

Fixed α = 0.02 B = 0.05

To allow for subgroup analysis, and to control for factors of age and function, as well as balancing of the number of participants between the groups at the three sites, we chose a sample size of n = 182 (i.e. 91/group), which corresponds to a minimal clinically important difference of 3. This difference was selected because it is plausible and feasible to achieve as shown by our pilot study, and it is associated with a three-point clinically important improvement [92]. This sample size (91/group) would also provide 80% power to detect time by treatment interaction. Thus, with 30 participants per group for each of the three sites, and to account for dropouts (20%), the planned total sample size is (30 × 3 sites) (20%) × 2 groups = 216 participants, or 108 participants per group.

#### Sampling for the qualitative data

The study will use purposeful criterion sampling to select persons participating in the FIT for FUNCTION program [93]. The sample size will be driven primarily by data saturation, as “collecting data until no new information is obtained” [94]. We will recruit 6–12 participants as recommended for phenomenological studies [95, 96], accounting for data saturation.

### Proposed methods for protecting against sources of bias

#### Contamination

Participants in the FIT for FUNCTION group will receive the intervention in a closed room at the YMCA three days/week. Persons in the control group who have a complimentary YMCA membership will self-determine the frequency of their attendance. Since the YMCA staff who interact with the intervention group will have no contact with the control group, and because the intervention will be delivered at a distance from routine programs, we anticipate there will be minimal opportunity for contamination.

#### Blinding

Independent assessors who are blind to group allocation will administer the study outcomes to the participants. The data analyst will also be blind to the group allocation.

#### Missing data

There are set timelines for administration of the outcomes and we will use multiple procedures to minimize missing data and loss to follow-up, such as repeated phone calls and mailed self-report assessments. In the pilot study, complete 12-week data were collected from 31 of 33 participants in the intervention group and 24 of 28 participants in the control group. Reasons for missing data included four participants who declined reassessment and two participants who we were unable to contact for follow-up. For the proposed trial, missing data will be assessed as to whether the pattern of missing scores is systematic or missing at random and we will use multiple imputation to perform the analyses.

### Data monitoring and auditing

This trial will not have a data monitoring committee and the study investigators will provide the auditing of the study since the estimated risk to the patients is low. The participants who undertake the research will be cleared to participate by a physician and determined to be medically stable and not at a high risk of mortality. The intervention of exercise and self-management and education have been studied with no serious adverse effects, so it is not expected that the participants will encounter any harm as a result of the intervention or control arm of the trial. There are no stopping rules or preliminary analyses planned.

### Analyses

The analysis and reporting of this trial will follow the CONSORT statement [97]. The baseline characteristics will be analyzed using descriptive statistics reported as mean (standard deviation [SD]) or median (first quartile [Q1], third quartile [Q3]) for continuous variables, depending on the distribution and count (percent) for categorical variables. All analyses of primary and secondary outcomes will follow the intention-to-treat principle. We will use multiple imputation to handle missing data [98]. The results will be reported as relative risk or odds ratio for binary outcomes or mean difference for continuous outcomes, with corresponding 95% CI and associated *p* value. All *p* values will be reported to three decimal places with those < 0.001 reported as *p* < 0.001. The criterion for statistical significance will be set at alpha = 0.05, adjusted using the Bonferroni method for multiple secondary outcomes. All analyses will be performed using Statistical Analysis Software 9.2 (Cary, NC, USA). Table 2 shows a summary of method of analysis for each outcome.

**Table 2:** Variables, measures, and methods of analysis

Variable	Outcomes	Outcome measure	Methods of analysis
Primary objective: To compare the effects of a 12-week community-based YMCA wellness program specifically designed for people with stroke vs a standard YMCA membership on community integration at 12 weeks and 24 weeks			
Community integration	Community integration	RNLI	Generalized linear models (GLM)
Secondary objective: To compare the effects of a 12-week community-based YMCA wellness program specifically designed for people with stroke vs a standard YMCA membership on the following variables:			
(a) Physical activity	Physical activity	RAPA, accelerometry	GLM
(b) Physical functioning	Lower extremity performance, strength, walking speed, balance	SPPB, dynamometer; 6MWT, BBS	GLM
(c) Health-related QOL	Health-related QOL	EQ-5D-5L	GLM
(d) Cardiovascular health	Cardiovascular health	BP, HR, BMI, waist and hip circumference	GLM
(e) Cardiovascular health	Lipids, glucose, and inflammatory markers	HDL and LDL cholesterol, triglycerides, glucose, and glycated hemoglobin	GLM
(f) Self-management	Self-management	PAM	GLM
(g) Healthcare utilization and costs	Healthcare utilization and costs	CRF	Mann–Whitney U test
Subgroup analyses: sex differences in response to treatment; age group differences response to treatment	Sex differences response to treatment; age group differences response to treatment	Interaction term sex × treatment group; interaction term age group × treatment group	Regression analysis with interaction test between each subgroup variable and treatment group evaluating the credibility of subgroup findings using previously published criteria
Sensitivity analyses: to evaluate the effect of group that adheres to treatment			Regression analysis

In all analyses, results will be expressed as estimate of effect, corresponding 95%, and associated *p* values

All tests will be one-sided using alpha = 0.05 level of significance in accordance with the non-inferiority hypotheses

*RNLI* Reintegration to Normal Living Index, *RAPA* Rapid Assessment of Physical Activity, *SPPB* Short Physical Performance Battery, *6MWT* 6-min walk test, *BBS* Berg Balance Scale, *EQ-5D-5L* European Quality of Life 5-Dimension Questionnaire, *BP* Blood pressure, *HR* Heart rate, *BMI* Body mass index, *HDL* High density lipoprotein, *LDL* Low density lipoprotein, *PAM* Patient activation measure, *CRF* Case report form

1. To assess the effect of the intervention on RNLI (research question 1), physical activity levels, physical functioning, QOL, cardiovascular health, and self-management (research question 2), we will estimate group, time, and group × time interaction effects using generalized linear models for continuous variables. We will include relevant baseline variables as covariates in the analysis that differ between groups at baseline, notably time since stroke (TSS) if there is a significant between-group difference. We will also include the baseline score for the specific measure of interest. For example, for research question 1, the baseline score of the RNLI would be included as a covariate for evaluating post-program changes.
2. Our hypothesis is that there will be reduced hospitalizations and physician visits for the intervention group compared to the control group (research question 3). Cost data are usually treated as non-parametric; therefore, a Mann–Whitney U test will be used to test relative ranks of participants between the groups on relative intervention cost.
3. We will use prognostic indicators from participants’ profiles to determine factors associated with the greater benefit from the FIT for FUNCTION program (research question 4). These factors will be identified by expanding the models for the treatment effects on RNLI and function as the dependent variables, and using prognostic variables of co-morbidities, illness severity, depressive symptoms, mobility impairment and cognitive status, education, previous level of activity, stroke classification, and interactions involving age and gender, as independent variables in the regression models.

#### Qualitative analysis

We will comprehensively explore the lived experience of participating in a community-based stroke wellness program) and community reintegration. Little is known about what community reintegration means to the person with stroke [99, 100]. An interpretive phenomenological approach will be utilized, following a constructivist paradigm [101]. Two reviewers will examine the transcripts and carry out independent, line-by-line analysis to generate initial codes, which will be finalized by consensus. Codes will then be consolidated into categories. The investigators will independently review each other’s coding of the transcripts to assure credibility. Next, we will extract quotes throughout the transcripts that correspond to the established codes and categories. Short summaries will describe the major ideas emerging from the data.

#### Cost-effectiveness analysis

The cost-effectiveness analysis will compare the incremental cost and effectiveness of the YMCA-based FIT for FUNCTION program with the standard YMCA membership for stroke survivors, from both the public healthcare payer’s perspective and the societal perspective. Effectiveness will be measured with QALY, using the utility values calculated from the EQ-5D-5L with duration. The main summary measure for the cost effectiveness analysis will be the incremental cost per QALY gained over the 24-week duration of the trial. Given that this cost-effectiveness analysis is conducted alongside the trial, non-parametric bootstrap will be used to calculate the 95% CI around the incremental cost per QALY ratio. The decision uncertainty will be presented using cost effectiveness acceptability curves, which show the probability of the intervention being cost-effective compared with the control group at specified societal willingness-to-pay for a QALY. The economic analysis will be conducted in using Statistical Analysis Software 9.2.

### Feasibility

The results of the pilot provided us with information about the feasibility of the project including the recruitment, randomization, the ability of this population to undertake and remain safely engaged in the program, and the suitability and responsiveness of the outcomes.

### Protocol modifications

We will communicate any changes to the study protocol to study investigators and the Hamilton Integrated Research Ethics Board and other research ethics boards (in writing) and electronically to the trial registry (www.clinicaltrials.gov). Participants will be contacted by phone to communicate any change in risk.

## Discussion

This proposed study will be the largest RCT examining the effectiveness of a community program for individuals with stroke. Additionally, as a RCT with broad study eligibility criteria, participants in the study will represent a wide spectrum of individuals living in the community with stroke, reflecting the heterogeneity of this population. Thus, if positive benefits are demonstrated, results will provide strong research evidence to support the implementation of structured, community-based exercise and education programs for a broad range of people living in the community with stroke. However, the rate of change is likely to be diverse, due to the wide age range and length of time since stroke, and this may limit the effect of the intervention. The advantage of the broad eligibility criteria is the generalizability of the results. We have expertise within our team to support the implementation of FIT for FUNCTION in communities from diverse regions; as a result of the pilot study, we developed strategies to address these challenges.

The eligibility, as noted, for this trial are very broad so there will be a heterogeneous sample of participants with a wide range of function, fitness, and ages who will potentially participate. This approach was intentional as this will be the target population who will receive the intervention through the YMCAs if it is a positive trial. However, this broad sampling approach may diminish the effect size. Although the participants of the trial need to gain medical clearance to participate, referral occurs by self-referral or by a health practitioner. This may result in some degree of sampling bias since this approach may self-select people who have had a previous propensity for exercise and engagement in positive health behaviors. Attrition maybe a factor due to age, reoccurrence of stroke, or other health issues. Absenteeism is often seasonal in this age group, where attendance rates may drop in the winter months when travel becomes increasingly challenging. Depression is often associated with stroke and may result in difficulties for participants remaining in the trial. Since participants are required to have completed bloodwork before having their baseline assessment and randomization, it is possible that persons who would usually join an exercise trial but who would not wish to undertake bloodwork will exclude themselves.

A limitation of the trial, as with most rehabilitation trials, is that it is not possible to blind the participants. Therefore, participants who are randomized to the control group may find it more difficult to adhere to exercise where there is less standardization and support compared to the intervention group. In addition, expanding this intervention to other YMCAs that have their own institutional idiosyncrasies may result in some site-specific effects, so we plan to examine the results of the trial by site. A further consideration is how time since stroke may influence the primary outcome of reintegration and may introduce bias. Persons who have low reintegration scores at one year, for example, may be different from participants with stroke who have low scores at 30–60 days which would result in the population at risk being different at the assessment time points. Persons who enter the trial at shorter time post stroke may have more spontaneous improvement and reintegrate earlier biasing the comparison to the null while persons who enter later may have more severe disease which will decrease the likelihood of a response in either group. To address this issue, we will adjust for time since stroke in our analyses and also use time since stroke to create subgroups to examine the outcomes.

A novel aspect of this study is it will be the first community-based trial to examine exercise-associated changes to cardiovascular risk factors. Despite the known increase in risk for recurrent events [4], cardiovascular risk factors are often poorly managed after stroke [13, 66]. The benefits of fitness training on walking ability are clearly established [102] but there is insufficient evidence that analogous improvements in cardiovascular risk factors can be observed. Few studies have included risk factors as outcomes and most have been conducted in research settings or in other controlled environments with smaller sample sizes. This study offers a large sample size to contribute to the state of the evidence related to changes in these risk factors following exercise and is the first community exercise trial to inform how to implement such strategies for secondary prevention within a sustainable community program model.

Ultimately, the results will help to inform how healthcare institutions and community agencies can work together to provide long-term exercise to persons of all ages following stroke and will increase the understanding about the degree of support and tailoring required to make these programs effective. It will also provide the foundation for future studies using factorial designs to more specifically examine effects of dosage and exercise types.

Another key innovation of the FIT for FUNCTION program is that it involves a unique and innovative collaboration through the LiveWell partners across healthcare, community, and academic centers. Our partners and collaborators include those who provide service delivery, those who coordinate regional stroke services, and from the Local Health Integrated Network who manage the overall integration of healthcare services. The YMCA delivers recreational services in all provinces and regions across Canada. This partnership will ensure that, if the results are positive, this model of care will be disseminated provincially and nationally within their organization and to other service providers. Each province and territory has a Stroke System and the YMCA is a national body, which will ensure that the impact from this trial is high.

### Anticipated results and research knowledge translation strategies

Results will be disseminated through professional organizations, the Ontario Ministry of Health and Long-Term Care, peer review conferences, journal articles, and special interest groups such as local and regional stroke recovery groups. Provincially, the Principal Investigators, Co-Investigators, Knowledge Users, and Collaborators on this current proposal will provide support and networking through Regional, Provincial, and National Stroke Networks such as the Heart and Stroke Foundation of Canada for other groups who wish to develop similar programs within their communities. The project results will be communicated at provincial and national stroke forums such as: The Annual Stroke Symposium Meeting of the Inter-Urban Stroke Academic Association; the Annual Stroke Collaborative; and the Canadian Stroke Congress. The LiveWell model and the study results will be also be disseminated provincially and nationally within the YMCA organization to stakeholders (healthcare professionals, healthcare administrators, and policy makers) from different provinces and territories.

## Trial status

Recruitment started in October 2014 and at the time of protocol submission, this study is recruiting participants. Recruitment is anticipated to be completed by December 2017.

## Additional files

Additional file 1: Table: Summary of community-based exercise programs for persons with stroke. (DOCX 20 kb)

Additional file 2: SPIRIT checklist. (PDF 55 kb)

Additional file 3: Figure: International classification of functioning, disability and health as applied to Fit for Function. (PDF 14 kb)

Additional file 4: Table: Details of the Fit for Function group exercise class. (DOCX 21 kb)

## Abbreviations

6MWT: 6-min walk test
BBS: Berg Balance Scale
BP: Blood pressure
CRF: Case report form
CVD: Cardiovascular disease
EQ-5D-5L: European Quality of Life 5-Dimension Questionnaire
HR: Heart rate
ICF: International Classification of Functioning, Disability and Health
LRC: Lead research coordinator
LWS: Living with Stroke
MoCA: Montreal Cognitive Assessment
PAM: Patient activation measure
QALY: Quality-adjusted life year
RAPA: Rapid Assessment of Physical Activity
RCT: Randomized controlled trial
RNLI: Reintegration to Normal Living Index
SE: Self-efficacy
SPPB: Short Physical Performance Battery
