# Supplementary material for: FIT for FUNCTION: study protocol for a randomized controlled trial
Source: Trials. 2018 Jan 15;19:39. doi: 10.1186/s13063-017-2416-3 (PMC5769391; doi:10.1186/s13063-017-2416-3)
Supplement: Supplementary file 1 — Table: Summary of community-based exercise programs for persons with stroke. (DOCX 20 kb) [file 13063_2017_2416_MOESM1_ESM.docx]

**Additional file 1:** Summary of Community-Based Exercise Programs for Persons with Stroke

| **Study and Design** | **Sample** | **Intervention** | **Institution** | **Outcome Measures** | **Results** |
| --- | --- | --- | --- | --- | --- |
| **Teixeira-Salmela** (1999)  Randomised  pre-test, post-test control group  design  CANADA | n=13  Mean age=67 years  Mean time since stroke=  7 years | 60-90 minutes  3x week for 10 weeks  Warm up  Aerobic exercises  Lower extremity  Muscle strengthening  Cool down  Control group received no intervention  Delivered by an exercise physiologist and a physiotherapist | Not given | Peak isokinetic torque of major muscle groups  Gait speed  Rate of stair climbing  Human Activity Profile(HAP)  Nottingham Health profile (NHP) | Exercise group showed significant improvement compared to controls  Peak isokinetic torque of major muscle groups p<0.004  Gait speed p<0.001  Rate of stair climbing p<0.001  HAP p<0.001  NHP p<0.001 |
| **Rimmer (2000)**  Pre-test, post-test lag control group design  USA | n=35  Mean age=  53 years  Approx.  6 months post-stroke  Predominantly African-American | 1 hr 3x week for 12 weeks  Cardiovascular,  Strength and flexibility  Delivered by an exercise physiologist,  A graduate student and 2 undergraduate interns and 3 volunteers | University based fitness centre | Grip strength  Body weight  Hamstring Flexibility  Shoulder flexibility  Peak V02 | Exercise group showed significant improvement compared to controls  Grip strength p<0.01  Body weight p<0.05  Hamstring Flexibility p<0.01  Peak V02 p<0.01  Shoulder flexibility NS  Grip strength NS  Waist to hip ratio NS |
| **Eng (2003)**  Single group  repeated measures design  CANADA | n=25 chronic stroke  Mean age=63 yrs  Mean time since stroke=  4 years | 8 week,  1 hr 3x week;  1 PT  2 kinesiologists  Balance, strength, functional strength and functional capacity | Research lab | Berg balance test (BBT)  12 Minute walk test (12MWT)  Stair climbing speed (SCS)  Reintegration into Normal Living Index (RNL)  Canadian Occupational Performance Measure (COPM) | Post intervention time effect for  BBT, 12Minute Walk Test (MWT)  SCS p<0.05  RNL(RNL) (NS)  COPM p<0.05 for performance and satisfaction  Program not sustained after study |
| **Study and Design** | **Sample** | **Intervention** | **Institution** | **Outcome measures** | **Results** |
| **Pang (2005)**  RCT, single blind  FAME  (fitness and mobility exercise program)  CANADA | n=63  Mean age=65 years  Mean time since stroke  =5 years | 19 week  1hr 3x week;  Cardiorespiratory,  Mobility, leg strength, balance and bone mineral density (BMD).  PT, OT and exercise instructor;  Control group rec’d upper extremity program | Community hall | Maximal oxygen consumption, 6MWT,  Isometric knee extension,  Berg Balance Test,  Physical Activity Scale for Individuals with Physical Disabilities;  BMD | Attended >80% sessions  Time-by-group interaction for  Maximal oxygen consumption p=0.03  6 MWT p=0.03  Paretic leg muscle strength  p=0.02  Paretic femoral neck BMD p=0.04  Program not sustained after study |
| **Leroux (2005)**  Pre-test/post-test design  CANADA | n=25  Mean age=68 years  Mean time since stroke  =5.5 years | 8 week 1hr 2x week  Balance, mobility, coordination, walking endurance and strength  Delivered by an exercise physiologist, undergraduate student and volunteer | Seniors Community Centre, Montreal | Stroke Assessment Impairment Set (SAIS)  Berg Balance Scale  The Step Test  Timed Up and Go  6MWT | Retention rate 65%  SAIS *  Berg Balance Test*  Step Test*  Timed up and Go*  *Sign diff between pre and post ex score p<0.008 |
| **Huijbregts (2008)**  MOST  Moving on After Stroke  Prospective longitudinal cohort design  CANADA | n=30  Mean age=68 years  Mean time since stroke=2 years | 17 2hr group based sessions  Intervention group  Self-management program and exercise program with land and Water exercise  Comparison group standard education program – Living with stroke (LWS)  Delivered by a PT assistant and 3 volunteers | Geriatric Care Facility, Toronto | Evaluation at end of program and 12 weeks follow-up  Activity Balance Scale (ABC)  Functional Independence Index (FIM)  Reintegration into Normal Living Index (RNL)  Geriatric Depression Score (GDS)  Chedoke-McMaster Stroke Assessment (CMSA) | 89% adherence rate for MOST and 90% for LWS  There were no between group differences on any of the outcomes.  Within group differences for the MOST group on  ABC p<0.005, FIM p<0.05, RNL p<0.05  Intervention group, within group differences  FIM p<0.05  MOST program sustained after study |

| **Study and Design** | **Sample** | **Intervention** | **Institution** | **Outcome measures** | **Results** |
| --- | --- | --- | --- | --- | --- |
| **Stuart (2009)**  Cohort study  Adaptive Physical Activity Community Based Exercise Program  ITALY | n=93  Mean age=68 years  Mean time post- stroke=4 years | 1 hr 3x week group based sessions  Classes 9-12 people)  Program aimed at muscle strengthening,  Joint flexibility,  Balance and cardiorespiratory function  Delivered by gym instructors trained and monitored by Physical therapists | Local gymnasiums | 6MWT  Short Physical Performance Battery (SPPB)  Berg Balance Test (BBT)  Stroke Impact Scale (SIS)  Barthel index (BI)  Hamilton Rating Scale Depression  Index of Caregivers Strain | 82% participants in the intervention group and 86% participants in the control group completed the study.  Between group differences at 6months in favour of the intervention group  6MWT p<0.0001  Motricity Index p<0.05  SPPB p<0.0004  BBT p<0.00015  Barthel Index p<0.385  Hamilton Rating Scale for Depression p<0.003  SIS participation p<0.00015  Caregiver strain p<0.09 |
| **Cramp (2010)**  Time series  Experimental Design  Baseline ax at 2 weekly intervals (A1); Exercise program 8-16 training sessions and repeat measurements at end of training (B); post training ax conducted 5-10 weeks after cessation of training (A2).  UK | n=18  Mean age=65 years  Mean time since stroke=  7 years | 60-90 minutes 2x weekly for 16 sessions  Group exercise low intensity progressive resistive exercise and functional tasks for lower limb  Delivered by fitness instructor with regular support from physiotherapists | Leisure centres | Maximal isometric and concentric knee flexor and extensor muscle groups were assessed.  Berg Balance Test  6 MWT  Barthel Index  Nottingham Extended Activities of Daily Living(NEADL) | Increase in:  Concentric knee extensor strength p<0.001 both limbs (BL)  Hip extensors p<0.001(BL)  Hip Abductors p<0.001(BL)  Ankle dorsiflexors p<0.002(paretic) and contralateral limb p<0.02  Ankle plantarflexors p<0.001(paretic)  And contralateral limb p<0.01  No significant increase in knee flexor strength in either limb  6MWT p<0.004  Berg Balance Score p<0.001  NEADL p<0.003 |
