# Supplementary material for: FIT for FUNCTION: study protocol for a randomized controlled trial
Source: Trials. 2018 Jan 15;19:39. doi: 10.1186/s13063-017-2416-3 (PMC5769391; doi:10.1186/s13063-017-2416-3)
Supplement: Supplementary file 3 — Figure: International classification of functioning, disability and health as applied to Fit for Function. (PDF 14 kb) [file 13063_2017_2416_MOESM3_ESM.pdf]

Additional file 3: International Classification of Functioning, Disability and Health as applied to Fit for Function

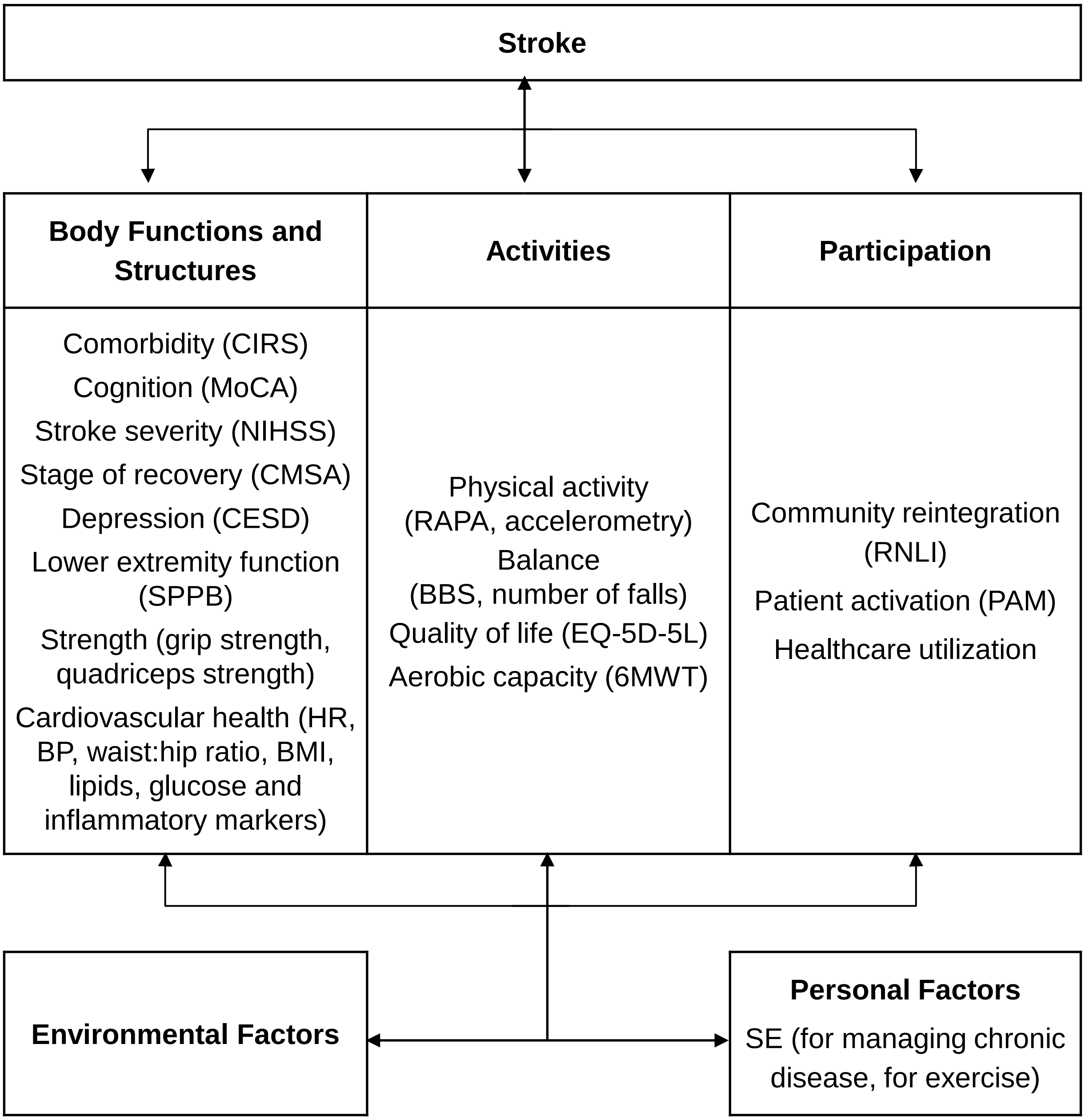

Legend: CIRS: Cumulative Illness Rating Scale; MoCA: Montreal Cognitive Assessment; NIHSS: NIH Stroke Scale; CMSA: Chedoke-McMaster Stroke Assessment; CESD: Centre for Epidemiologic Studies Depression Scale; SPPB: Short Physical Performance Battery; HR: Heart rate; BP: Blood pressure; BMI: Body-mass index; RAPA: Rapid Assessment of Physical Activity; BBS: Berg Balance Scale; EQ-5D-5L: European Quality of Life 5-Dimension Questionnaire; 6MWT: 6-Minute Walk Test; RNLI: Reintegration to Normal Living Index; PAM: Patient Activation Measure; SE: Self-efficacy.
