# Supplementary material for: FIT for FUNCTION: study protocol for a randomized controlled trial
Source: Trials. 2018 Jan 15;19:39. doi: 10.1186/s13063-017-2416-3 (PMC5769391; doi:10.1186/s13063-017-2416-3)
Supplement: Supplementary file 4 — Table: Details of the Fit for Function group exercise class. (DOCX 21 kb) [file 13063_2017_2416_MOESM4_ESM.docx]

**Additional file 4:** Fit For Function: Group Exercise Class

| **Warm Up:** | | | | | |
| --- | --- | --- | --- | --- | --- |
| 1. Sitting/standing posture: take a minute to focus on posture (sitting and standing). Encourage this posture throughout class, especially remaining centered. | | | | | |
| 1. Deep breathing…progress to… hand(s) on ribs/abdomen… raising pain free arm(s). | | | | | |
| 1. Upper extremity warm up: shoulders, elbows, wrists and fingers.  - Strong arm should assist weak as needed. - An assisted arm should not be moved above shoulder level. - If arm is painful it should not be moved above shoulder level or into pain. - Encourage thumbs up position with shoulder movements.   - Ideas: shoulder shrugs/rolls (fwd./back), front arm raises, palms up/down, hands to shoulders (elbow flexion, use hands together if needed) and then to knees, wrist circles (hands together if needed), palms together spread fingers (or spread tight fingers onto thigh with strong hand), pass object around (maybe more than one). | | | | | |
| 1. Ankle mobility:  - If weak foot unable, then use strong leg and imagine weaker one moving, remove top strap of brace if necessary (replace when finished) and slide foot under chair with heel down to stretch. - Ideas: toe tapping, heel tapping, heel/toe rocking, circles, straighten knee and then move ankle, slide foot under the chair keeping heel down. | | | | | |
| 1. Marching on the spot:   Support options:   - Stand behind the chair and use 1 or 2 hands for support - Turn sideways and hold on with strong hand - No support, swinging arms naturally | | | | | |
| *Low level* | *Intermediate Level* | | | | *High level* |
| Marching | Change height of steps  Change width of steps (step wide/narrow) | | | | Tapping opposite knee  Clap under knee |
| **Cardiovascular Conditioning:** | | | | | |
| **Walking: 10 minutes** | | | | | |
| Progression ↓ | | | Variations | | |
| Inside lane, with/without gait aid  Outside lane  Add circles around the chairs  Carry a weight  Add arm movements with weights (elbow flexion)  Cycle on bike | | | Call out stop/start, or change direction  Look side to side while walking  Vary speed on a length or lap  Step over lines on the floor | | |
| **Group Task-Oriented Strengthening** | | | | | |
| **Exercise 1: Sit to Stand: 3 x 10 if able** | | | | | |
| Progression ↓ | | | Variations | | |
| Stand up/ sit down using arm rest(s)  **Stand up/sit down with hand(s) on chair**  Stand up/sit down with hand(s) on knee  Stand up/sit down with no hands  Place weaker leg back further when standing/sitting | | | Increase speed  Hold an object in hands  Lightly touch buttocks down on an object and stand again | | |
|  |  |  | Adaptation | | |
|  |  |  | Use a cushion on the chair if the person has difficulty.  May need to place chair against wall for stability | | |
| **Exercise 2: Heel Raises: 3 x 10 if able** | | | | | |
| Progression ↓ | | | Variations | | |
| Push up onto toes, using chair for support  Stand on one leg, push up onto toes | | | Increase speed  Add alternate toe lifts | | |
|  |  |  | Adaptation | | |
|  |  |  | Can be done in sitting | | |
| **Mobility Circuit** | | | | | |
| **Station 1: Stand, Walk and Carry**  Stand from chair, walk behind to the basket, pick up an object and carry it to the opposite side, placing it in that basket and sitting in that chair. | | | | | |
| *Low level* | | *Intermediate level* | | *High level* | |
| Fill a bag with a few objects and carry it  Pick up and put down 3 objects and walk with just the third | | Walk sideways (side step) with the object.  Carry the whole basket with one or two hands or carry a large ball.  Carry a tray with two hands; add an object to balance on the tray.  Carrying weights in one/two bags.  While carrying objects, step through non-slip rings on floor or pick up rings on the way. | | Add a non-slip mat between the chairs to walk over top of (caution of foot catching).  Pick up a large ball from the floor, place it at waist level against a wall, and then lift to shoulder level, waist level and back to the floor, repeating several times.  Carry an object or ball, marching with high knees, tapping knees with object each step.  Carry an object to the wall and hold it while doing wall slides (5-15 reps) and return to chair. | |
| **Station 2: Stepping 3x10 reps**  Support should be set up individually either from a chair, bar or wall as needed. | | | | | |
| *Low level (with support)* | | *Intermediate level* | | *High level* | |
| Tap ups (front)  Tap ups (side) | | Step ups (front)  Step ups (side) | | Step up and over- front  Step up and over- side | |
| Progression: Higher level of step, quicker speed | | | | | |
| Variation: For knee joint problems just do tap ups or keep repetitions and height low | | | | | |
| **Station 3: Balance**  Use support from a bar, chair, wall or bike. Sit if needed. | | | | | |
| *Low level* | | *Intermediate level* | | *High level* | |
| Standing: eyes closed  Standing: look over shoulders  Heel toe rocking  Backward walking  Sidestepping  Step out and together | | + step stance  + step stance  Heel toe rock on bosu  Tandem forward  Braiding  Step side/front together | | + tandem stance  + tandem stance  Step on bosu  Tandem fwd/backward  Braiding, back/front  + no support and quickly | |
| **Station 4: Wall Target Reaching:** | | | | | |
| *Low level* | | *Intermediate level* | | *High level* | |
| Wide stance  Weight shift side to side | | Feet together  Step stance | | Unipedal stance  Tandem stance | |
| Adaptation: For lower level stand closer or sit in a chair. | | | | | |
| **Cool Down:** | | | | | |
| 1. Controlled deep breathing (as in warm up) 2. Hamstring stretch 3. Trunk rotation stretch 4. Calf stretch- loosen top of AFO strap for exercise and then replace | | | | | |
